# Clinical trial experience with CA4P anticancer therapy: focus on efficacy, cardiovascular adverse events, and hypertension management

**DOI:** 10.1186/s40661-017-0058-5

**Published:** 2018-01-05

**Authors:** Rachel Grisham, Bonnie Ky, Krishnansu S. Tewari, David J. Chaplin, Joan Walker

**Affiliations:** 1 Memorial Sloan Kettering Cancer Center and Weil Cornell Medical College, New York, NY USA; 2 Perelman School of Medicine, University of Pennsylvania, Philadelphia, PA USA; 3 Division of Gynecologic Oncology, University of California Irvine, Orange, CA USA; 4 Mateon Therapeutics, South San Francisco, CA USA; 5 The Stephenson Cancer Center, University of Oklahoma, Oklahoma City, OK USA

**Keywords:** Bevacizumab, Blood pressure, CA4P, Cardiovascular, Combretastatin A4-phosphate, Focus, Fosbretabulin, Hypertension

## Abstract

Combretastatin A4-phosphate (CA4P) is a vascular-disrupting agent (VDA) in clinical development for the treatment of ovarian and other cancers. In contrast to antiangiogenic agents, such as bevacizumab, which suppress the development of new tumor vasculature, VDAs target established tumor vasculature. These differing but complementary mechanisms of action are currently being explored in clinical trials combining CA4P and bevacizumab. Clinical experience to date has highlighted an important need to better understand the cardiovascular adverse events of CA4P, both alone and in combination with antiangiogenic agents, which can also be associated with cardiovascular adverse events.

An acute but transient increase in blood pressure is often the most clinically relevant toxicity associated with CA4P. Increases in CA4P-related blood pressure typically occur 0.5 to 1 h after initiation of the 10-min infusion, peak by 2 h, and return to baseline 3 to 4 h after the infusion. Post-infusion increases in blood pressure are likely to recur in subsequent treatment cycles; however, the severity does not appear to increase with successive cycles. Other cardiovascular adverse events, such as transient, predominantly grade 1–2 tachycardia, bradycardia, QTc prolongation, and in rare cases myocardial ischemia, have also been observed with CA4P but at markedly lower frequencies than hypertension.

The clinical trial experience with CA4P suggests that cardiovascular assessment of patients prior to CA4P treatment and careful management of blood pressure during CA4P treatment can largely mitigate the risk of cardiovascular adverse events. Accordingly, we have developed a blood pressure management algorithm for use in the ongoing phase II/III FOCUS study of the triple combination of CA4P with physician’s choice chemotherapy and bevacizumab.

## Introduction

Tumor vasculature is a long-established target of anticancer therapy [1]. Vascular-targeted anticancer therapies include two broad categories of agents with complementary mechanisms of action [2]: antiangiogenic agents (AAs), which prevent tumor neovascularization by inhibiting vascular endothelial growth factor and other pro-angiogenic factors, and vascular disrupting agents (VDAs), which destroy established tumor vasculature. The most extensive preclinical and clinical VDA data profile is associated with the tubulin-binding VDA, combretastatin A4-phosphate (CA4P) [3]. CA4P binds to tubulin, at or close to the colchicine binding site, causing disruption of the tumor endothelial cell cytoskeleton and junctions between endothelial cells. This results in endothelial cell shape change, leaky vessels, congestion within the blood vessel lumen, cessation of blood flow, and ultimately tumor necrosis [4, 5]. The preferential targeting of tubulin-binding VDAs to tumor vasculature as opposed to that of normal vasculature is due to the relative immaturity and instability of tumor vasculature [6].

VDAs, including CA4P, demonstrate limited single-agent antitumor activity [7, 8]. Preclinical data indicate that this limited single-agent activity is attributable to a remaining viable rim of tumor cells that are supported by oxygen and nutrients from the surrounding normal vasculature [4, 7–9]. Without additional treatment the tumor can rapidly revascularize. It has been proposed that the combined use of AAs and VDAs might circumvent this issue since AAs can inhibit this neovascularization while VDAs target the already formed, but abnormal, tumor vasculature inducing extensive cellular necrosis at the tumor core [10, 11]. This combined approach is being studied for the treatment of ovarian cancer (OC) in a phase II/III trial of physician’s choice chemotherapy and bevacizumab with or without CA4P and in a phase I/II trial of CA4P plus pazopanib. Clinical experience with CA4P to date suggests that the most frequently occurring adverse events (AEs) associated with CA4P treatment are acute but transient blood pressure (BP) increases. Hypertension (HTN) is also associated with bevacizumab [12], pazopanib [13], and other AAs [14]. Thus, establishing an understanding of the cardiovascular safety profile of CA4P–antiangiogenic combination therapies is an important step in the clinical development of these therapeutic regimens. This article reviews the cardiovascular safety profile of CA4P as a single-agent and in combination regimens and reports a BP management algorithm developed by an expert panel based on these data and clinical experience.

## Review

### CA4P clinical studies: Efficacy

CA4P has been studied in phase II trials in several tumor types [15–19]. In the phase II FALCON study, 63 patients with chemotherapy-naive stage IIIb/IV non–small cell lung cancer (NSCLC) were randomized to CP and bevacizumab with or without CA4P (60 mg/m^2^) [16]. CA4P did not confer a significant survival benefit (median OS 13.6 vs 16.2 months; HR = 1.06 [95% confidence interval [CI], 0.55–2.03]), but it was associated with a substantial increase in response rate compared with the control arm (50% vs 32%, respectively). Interestingly, post hoc analyses showed a trend toward longer survival with CA4P–CP–bevacizumab compared with CP–bevacizumab in patients with tumors >10 cm (median OS 14.2 vs 11.0 months; HR = 0.67 [95% CI, 0.26–1.70]).

A single-arm, phase II study evaluated the combination of CA4P (63 mg/m^2^) and CP in 44 patients with OC that had recurred <6 months after platinum therapy [17]. A confirmed response (all partial responses) was observed in 5 of 37 (13.5%) patients who were evaluable by Response Evaluation Criteria in Solid Tumors. The response rate was 34% in the 38 patients evaluable by Gynecologic Cancer InterGroup CA 125 criteria. GOG-0186I—a randomized, phase II study—evaluated CA4P (60 mg/m^2^) in combination with bevacizumab versus bevacizumab alone in 107 patients with recurrent OC [18, 19]. Patients in the CA4P arm had a near-3-month PFS benefit (median PFS, 7.3 vs 4.8 months; HR = 0.69 [90% CI 0.47–1.00], *p* = 0.05) [18]. Notably, in the 81 patients with measurable disease, the PFS benefit was enhanced in those treated with CA4P + bevacizumab compared with those receiving bevacizumab alone (9.8 vs 6.1 months; HR = 0.60, *p* = 0.027) [19]. The PFS benefit was further enhanced in patients with tumors greater than the median size (>5.7 cm) for the study population with measurable disease at baseline (10.5 vs 4.3 months; HR = 0.554, *p* = 0.071), again demonstrating a greater benefit in patients with larger tumor size [19]. Together, the clinical data support the potential for CA4P in the management of varied cancer types, with clear signals in recurrent OC as well as ATC and NSCLC [15–19].

### CA4P clinical studies: Safety

In the studies to date, CA4P has been studied as monotherapy and in combination with other treatments, such as antiangiogenic therapy (eg, bevacizumab) and chemotherapy (eg, CP). CA4P has been generally well tolerated with the most notable AEs across studies being hematologic toxicity, tumor pain, and HTN.

#### Cardiovascular AEs

**Fig. 1:**
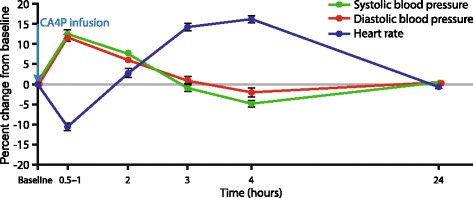
Time course of mean [20, 21] and median [17] heart rate changes from baseline, and mean [16, 20, 23] and median [17] blood pressure changes [17, 20, 23] from baseline in combretastatin A4-phosphate (CA4P) studies reporting such data. CA4P infusion occurred at time 0. Means of published data points are shown. Standard error of the mean is indicated with error bars

Cardiovascular AEs have been the most frequently and consistently reported AEs across CA4P studies (Table 1). By far, the most common of these has been an acute, transient increase in BP (see Table 1). Typically, in studies of single-agent CA4P or CA4P + CP, increases of approximately 10% to 15% above baseline were seen at 0.5 to 1-h post-infusion. These resolved by 3 to 4 h post-infusion [17, 20, 21] (Fig. 1).

**Table 1:** Adverse events related to the cardiovascular system in studies of CA4P

Study		Phase II single-arm CA4P (63 mg/m^2^) + CP (*n* = 44) [17]	Phase II, two-arm^a^ Bev (15 mg/kg) + CA4P (60 mg/m^2^) (*n* = 54) OR Bev (*n* = 53) [18, 24]	Phase II single-arm CA4P (60 mg/m^2^) + CP (*n* = 51) [15]	Phase II, two-arm^a^ CP + Bev (15 mg/kg) + CA4P (60 mg/m^2^) (*n* = 31) OR CP + Bev (*n* = 29) [16]	Phase I single-arm CA4P (doses from 18 mg/m^2^ to 90 mg/m^2^) (*n* = 25) [21, 22]^b^	Phase I single-arm CA4P (doses from 5 mg/m^2^ to 114 mg/m^2^) (*n* = 34) [20]	Phase I single-arm CA4P (doses from 45 mg/m^2^ to 63 mg/m^2^) + Bev (10 mg/kg) (*n* = 15) [23]
Tumor type		Platinum-resistant ovarian cancer	Recurrent ovarian cancer	Anaplastic thyroid cancer	Stage IIIb/IV NSCLC	Solid tumors	Solid tumors	Solid tumors
Hypertension, *n* (%)	All-grades	10 (23)	32/53 (60)	17 (33)	17 (55)	1 (4)	12 (35)	11 (73)
	Grade ≤ 2	10 (23)	NR	15 (29)	NR	1 (4)	12 (35)	11 (73)
	Grade 3	0	19 (35)	2 (4)	NR	0	0	0
	Grade 4			0	NR	0		0
Tachycardia, *n* (%)	All-grades	15 (34)	2 (4)	6 (12)	8 (26)	NR	19 (56)	1 (7)
	Grade ≤ 2	14 (32)	NR	6 (12)	NR	NR	19 (56)	1 (7)
	Grade 3	1 (2)	NR	0	NR	NR	0	0
	Grade 4		NR	0	NR	NR		0
Bradycardia, *n* (%)	All-grades	1 (2)	3 (6)	2 (4)	4 (13)	NR	8 (24)	NR
	Grade ≤ 2	1 (2)	NR	1 (2)	NR	NR	8 (24)	NR
	Grade 3	0	NR	1 (2)	NR	NR	0	NR
	Grade 4		NR	0	NR	NR		NR
Hypotension, *n* (%)	All grades	2 (5)	3 (6)	NR	NR	NR	6 (18)	NR
	Grade ≤ 2	2 (5)	NR	NR	NR	NR	6 (18)	NR
	Grade 3	0	NR	NR	NR	NR	1 (3)	NR
	Grade 4		NR	NR	NR	NR		NR
Arrhythmia, *n* (%)	All grades	2 (5)	2 (4)	0	NR	2 (8)	NR	1 (7)
	Grade ≤ 2	1 (2)	NR	0	NR	2 (8)	NR	0
	Grade 3	1 (2)	NR	0	NR	0	NR	1 (7)
	Grade 4		NR	0	NR	0	NR	0
Myocardial ischemia, *n* (%)	All grades	1 (2)	NR	2 (4)	2 (6)	2 (8)^c^	NR	NR
	Grade ≤ 2	1 (2)	NR	2 (4)	0	1 (4)	NR	NR
	Grade 3	0	NR	0	2 (6)	0	NR	NR
	Grade 4		NR	0	0	1 (4)^c^	NR	NR
>QTc, *n* (%)	All grades	2 (5)	NR	8 (16)	NR	7 (28)	0	NR
	Grade ≤ 2	2 (5)	NR	6 (12)	NR	7 (28)	0	NR
	Grade 3	0	NR	2 (4)	NR	0	0	NR
	Grade 4		NR	0	NR	0	0	NR
AV block, *n* (%)	All grades	NR	NR	1 (2)	NR	NR	NR	NR
	Grade ≤ 2	NR	NR	1 (2)	NR	NR	NR	NR
	Grade 3	NR	NR	0	NR	NR	NR	NR
	Grade 4	NR	NR	0	NR	NR	NR	NR

^a^In two-arm studies, adverse events are shown only for the CA4P-containing arms

^b^These manuscripts describe the same patient cohort so safety data were combined

^c^Grade 4 myocardial ischemia occurred in a patient receiving 90 mg/m^2^ of CA4P

*AV* atrioventricular, *Bev* bevacizumab, *CA4P* combretastatin A4-phosphate, *CP* carboplatin and paclitaxel, *MI* myocardial infarction, *NR* not reported, *NSCLC* non–small cell lung cancer

The HTN observed in these studies was predominantly grade 1–2 [15, 17, 20–22]. In the FACT study in ATC, grade 1–2 HTN was increased with the addition of CA4P to CP (29.4% vs 0% with CP) [15]. The incidence of grade 3 HTN was similar with CP with and without CA4P (3.9% and 4.2%, respectively), and no grade 4 HTN was observed. In the single-arm, phase II study of CP and CA4P in patients with recurrent OC, grade ≤ 2 HTN was observed in 23% of patients, but no grade 3 or 4 HTN was observed [17]. Notably, there was no cumulative hypertensive effect demonstrated with consecutive treatment cycles.

Bevacizumab is commonly associated with sustained HTN. In a meta-analysis of 12,656 patients treated with bevacizumab, HTN was demonstrated in 23.6% of patients, including 7.9% who had HTN categorized as grade ≥ 3 [12]. Given the established association of HTN and bevacizumab, the incidence of HTN in patients receiving CA4P in combination with bevacizumab is of particular interest. Consistent with studies of single-agent CA4P or chemotherapy + CA4P, increases in transient BP were seen in patients receiving CA4P + bevacizumab. The time course was similar to prior studies. Increases in BP typically occurred within the first 30 min to 1 h after infusion, persisted for approximately 2 h, and returned to baseline by 4 h [16, 23]. The addition of bevacizumab to CA4P appeared to increase the magnitude of the increases in BP during the first three bevacizumab-containing cycles but not in subsequent cycles. Systolic and diastolic BP were increased by approximately 10% in these combination-treatment cycles compared with values with CA4P alone in cycle 1 [23]. Notably, there was no grade 3 or higher HTN. The addition of bevacizumab to CA4P also appears to increase the incidence of HTN overall. In the FALCON study of CA4P in combination with CP–bevacizumab in patients with NSCLC, all-grade HTN was increased in the CP–bevacizumab–CA4P arm relative to the CP–bevacizumab arm (55% vs 45%) [16]. In the GOG-0186I trial, in which patients were randomized to bevacizumab with and without CA4P, grade ≥ 3 HTN was increased with bevacizumab–CA4P (35% vs 20% with bevacizumab alone; relative risk, 1.77 [95% CI, 0.90–3.45]) [18]. Because of the increased incidence of HTN reported with CA4P + bevacizumab, active monitoring of BP and management in clinical trials of this combination is essential.

Other cardiovascular AEs, such as tachycardia, bradycardia, and QTc prolongation, have also been observed with CA4P [15–18]. In phase II studies to date, tachycardia and bradycardia have been reported in 4% to 34% and 2% to 13% of patients, respectively (see Table 1) [15–17]. With the exception of one case of grade 3 tachycardia [17] and one case of grade 3 bradycardia [15], all events have been grade 1 or 2. The heart rate changes are typically characterized by a decrease in heart rate within the first hour after infusion followed by an increase between 3 and 4 h post-infusion, and a return to baseline by 24 h [17, 20–22]. The typical time course of heart rate changes following CA4P administration are shown in Fig. 1.

QTc prolongation has also been reported in CA4P studies; however, to date, a dedicated QTc study has not yet been performed. In the FACT study in ATC, all-grade and grade 3 QTc prolongation were reported for 16% and 4% of patients receiving CP–CA4P, respectively, and one patient discontinued treatment because of QTc prolongation. There were no reports of QTc prolongation in the control arm; however, since electrocardiograms were not routinely collected in the control arm, the rate of QTc prolongation in the control arm may have been under-reported [15]. QTc prolongations were also reported in two single-arm studies of CA4P, but all were grade 1 or 2 [17, 21, 22] and deemed clinically insignificant [21, 22].

To date, across eight studies, seven patients administered CA4P-containing regimens have experienced myocardial ischemia [15–17, 21, 22]. In the phase II FALCON study, two patients in the CA4P–CP–bevacizumab arm, both with a history of HTN, experienced three episodes of grade 3 myocardial ischemia, which resulted in treatment discontinuation [16]. One event occurred concurrently with a post-CA4P infusion BP increase and another occurred during the bevacizumab infusion, 24 h after the CA4P infusion. Three patients in the other phase II studies experienced myocardial ischemia, but it was asymptomatic and grade 1 in two patients [15] and grade 1–2 in the other [17]. In a single-agent CA4P dose-finding study, two patients (one treated with 60 mg/m^2^ CA4P and one with 90 mg/m^2^ CA4P) had myocardial ischemia (one grade 2; one grade 4) [22]. The patient treated with 60 mg/m^2^ CA4P had a grade 2 event and subsequently was found to have coronary artery disease, which was treated with angioplasty. The patient recovered fully with no further cardiac issues during the 11 months he was followed after treatment discontinuation [21, 22]. The patient treated with 90 mg/m^2^ of CA4P experienced grade 4 myocardial ischemia secondary to coronary artery vasospasm. An electrocardiogram was performed, and it was consistent with myocardial infarction. Cardiac catheterization showed subtotal stenosis. Serial troponin levels were normal. The patient recovered the same day with a normal electrocardiogram and left ventricular ejection fraction [21, 22].

#### Hematologic toxicity

The rate of all-grade hematologic toxicity increased with the addition of CA4P to chemotherapy [15] (all-grade leukopenia CP 4% vs CP + CA4P 41%; all-grade neutropenia CP 21% vs CP + CA4P 57%) and to chemotherapy + bevacizumab [16] (all-grade leukopenia CP + bevacizumab 24% vs CP + bevacizumab + CA4P 45%; all-grade neutropenia CP + bevacizumab 48% vs CP + bevacizumab + CA4P 81%). However, in GOG-0186I, in which neither treatment arm contained chemotherapy, the rates of all-grade leukopenia (21% vs 15%) and all-grade neutropenia (13% vs 15%) were similar in the bevacizumab alone and the CA4P + bevacizumab study arms [24].

#### Tumor pain

Grade 3–4 tumor pain was reported in the CA4P arm of the FACT study but not in the control arm (6% vs 0%) [15]. Grade 3–4 pain was also observed in 18% of patients with recurrent OC treated with CP and CA4P in the single-arm phase II study [17]. In most cases, tumor pain occurred within an hour after CA4P infusion and resolved with pain medication. No patient discontinued treatment due to pain. Tumor pain has also been observed in several phase I studies of CA4P [20, 21, 25]. This tumor pain was observed more frequently in patients with OC who responded to treatment (67% vs 48%), though this correlation was not statistically significant [17]. A potential relationship between tumor pain and heart rate and/or BP should not be overlooked because tumor pain may exacerbate these AEs. Active management with pain medication is recommended.

### Management of CA4P-induced BP increases: Best practice

Preclinical data demonstrate that the cardiovascular AEs associated with CA4P can be prevented by pretreatment with calcium channel blockers, suggesting that CA4P does not induce direct cardiotoxic effects [26]. Administration of CA4P to hypertensive rats resulted in a significant increase in mean arterial pressure and, in a number of animals, circulating troponin I. The calcium channel blockers diltiazem and nicardipine completely eliminated the hypertensive effects and pretreatment with diltiazem prevented increases in serum troponin in these animals [26]. Similarly, administration of the tubulin-binding VDA ZD6126 has been shown to elevate BP in rats, increase circulating troponin, and induce left ventricular myocardial fiber necrosis [27]. These effects were all blocked when animals were pretreated with a calcium channel blocker in combination with a beta blocker to prevent HTN [27].

Preclinical and clinical data suggest that CA4P-induced BP increases are a compensatory response to an increase in peripheral resistance [28, 29]. In a preclinical study in rats, which measured blood flow using a radiolabel and quantitative autoradiography, arterial BP was increased at 1 and 6 h after CA4P administration, and by 6 h, mean tumor blood flow was reduced by more than 100-fold [28]. Blood flow was also reduced in other tissues, most notably, the spleen (seven-fold decrease). In a clinical study using positron emission tomography to evaluate 13 patients treated with a radiolabel and CA4P, mean tumor perfusion was reduced (−49%), beginning 30 min after administration of CA4P [29]. Decreases were also observed in spleen perfusion (−35%) and kidney perfusion (−6%).

Because of their transient nature, the underlying pathophysiology of the BP increases associated with CA4P appear to differ from that of the sustained BP increases observed with bevacizumab therapy [30]. This supports different management strategies for HTN associated with CA4P and bevacizumab. The clinical trial experience with CA4P lends support to careful patient selection prior to CA4P therapy along with cardiovascular assessment and careful management of BP during and after CA4P infusion.

**Fig. 2:**
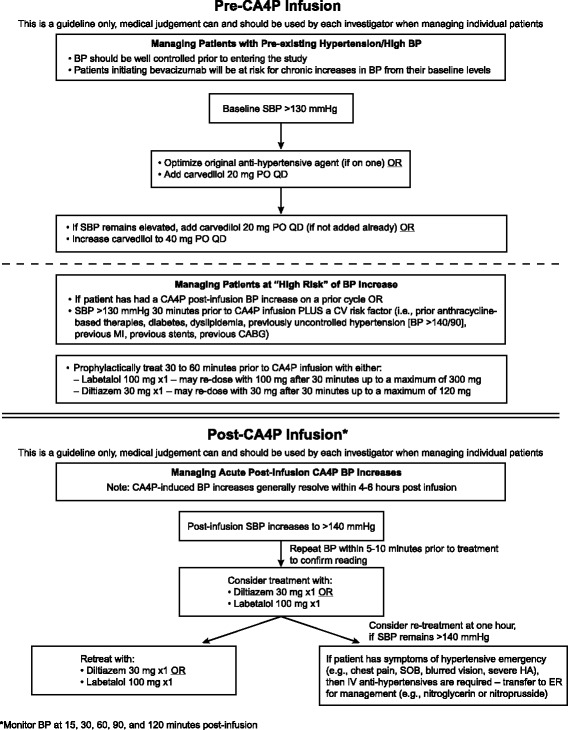
Combretastatin A4-phosphate (CA4P) blood pressure management algorithm. BP = blood pressure; CV = cardiovascular; ER = emergency room; HA = headache; PO = orally; QD = daily; SBP = systolic blood pressure; SOB = shortness of breath

To optimize the cardiovascular risk profile of CA4P therapy, an expert panel was convened to develop a BP management algorithm for use in the phase II/III FOCUS study [31] (Fig. 2). The panel agreed that different treatment strategies should be used for patients with baseline HTN (defined for this study as systolic BP >130 mmHg) and those without. For those with baseline HTN, the panel recommended that their current antihypertensive medication be optimized. Carvedilol was recommended as an initial agent for BP control because it acts at both alpha- and beta-adrenergic receptors, is fairly well tolerated, and combines well with other antihypertensive agents. Moreover, a beta blocker strategy would be beneficial in the setting of myocardial ischemia, and there is a longstanding literature suggesting a cardioprotective and beneficial cardiac remodeling effect with carvedilol [32, 33]. The most common side effect associated with carvedilol is dizziness [34, 35]. Of note, carvedilol carries an FDA black-box warning against abrupt cessation of treatment in patients with coronary artery disease as this can exacerbate angina or result in myocardial infarction or ventricular arrythmia. Therefore, it is critical that cessation of therapy is strictly monitored and carried out in accordance with the prescribing information [34, 35].

For patients without baseline HTN, the panel recommended that an evaluation of cardiovascular risk factors be performed before therapy is started. Cardiovascular risk factors should include prior anthracyclines, diabetes, dyslipidemia, prior uncontrolled HTN, previous myocardial infarction, previous stents, or previous coronary artery bypass grafting. Consideration of patients as “high risk” is recommended if patients had a previous CA4P-induced BP increase or if they have systolic BP >130 mmHg 30 min prior to CA4P infusion plus a cardiovascular risk factor as defined above.

The panel recommended that BP be monitored frequently after infusion at intervals of 15, 30, 60, 90, and 120 min. Should systolic BP reach 140 mmHg, treatment with diltiazem or labetalol with continuous monitoring was advised. If systolic BP remains at or above 140 mmHg at 1 h, retreatment with diltiazem or labetalol is advised or, if patients experience symptoms of hypertensive emergency, patients should be transferred to emergency room care.

The recommended antihypertensive agents were chosen for several reasons. As discussed earlier, CA4P-induced BP increases typically begin 0.5 to 1 h post-infusion and resolve by 3 to 4 h post-infusion [17, 20–22]. Thus, to avoid hypotension, it is important that the antihypertensive agents used to manage these BP increases have a quick onset of action and are short-acting. Additionally, agents that could result in reflex tachycardia should be avoided, given the risk of CA4P-associated tachycardia.

The alpha- and beta-adrenergic receptor blocker, labetalol, and the calcium channel blocker diltiazem have pharmacokinetic profiles that mesh well with the time course of CA4P-induced BP increases. The onset of the antihypertensive effect of oral labetalol is between 30 and 120 min, the maximum effects are observed within 1 to 3 h after administration and the plasma half-life is 6 to 8 h [36]. The onset of action for immediate release diltiazem is between 30 and 60 min, peak plasma levels are observed 2 to 3 h after administration and the plasma half-life is 3.5 h [37].

Because patients with cancer are typically being treated with multiple medications, the potential for drug–drug interactions and additional side-effects are another key consideration when developing an antihypertensive strategy. Labetalol has a relatively low-risk of drug–drug interaction [36]; however, diltiazem is metabolized by CYP34A so care should be taken when prescribing it to patients taking other drugs that interact with CYP34A [37]. Side-effects of labetalol include, dizziness, nausea, and fatigue [36] and side effects of dilitiazem include edema, headaches, nausea, and dizziness [37]. Labetalol may have an added advantage in that some data suggest that tumor cells express beta 1-, beta 2- and beta 3-adrenergic receptors and that these receptors can mediate tumor cell proliferation and facilitate metastasis [38, 39]. Some retrospective studies suggest that blockade of these receptors is associated with improved outcomes in patients with cancer [40–42]. However, other studies have not found such an association [43, 44].

## Conclusions

VDAs, including CA4P, disrupt the existing tumor vasculature within the interior of tumors, a region that is often resistant to standard therapies, such as chemotherapy and radiation, and may have particular benefit in patients with bulky disease. CA4P has a contrasting and complementary activity compared with AAs, such as bevacizumab, and the combination of these agents is supported both by a mechanistic rationale and promising clinical data. While CA4P and bevacizumab are associated with toxicity profiles dominated by BP effects, the agents appear to be able to be used safely in combination with appropriate patient selection and active monitoring and treatment. While bevacizumab primarily causes sustained HTN that requires modulation of a daily antihypertensive regimen, the BP surges seen with CA4P most commonly resolve within hours after drug administration and are best treated either with pretreatment in selected high-risk patients or with immediate administration of antihypertensive therapy at the time of the first BP increase. Frequent BP monitoring is essential immediately after CA4P administration to mitigate associated complications. It is anticipated that the ongoing FOCUS phase II/III trial of physician’s choice chemotherapy, bevacizumab, and CA4P in patients with platinum-resistant OC, which employs a proactive BP management strategy, will provide key data on the efficacy and safety of triple combination therapy [31].

## Abbreviations

AA: Antiangiogenic agent
AE: Adverse event
ATC: Anaplastic thyroid cancer
BP: Blood pressure
CA4P: Combretastatin A4-phosphate;
CI: Confidence interval
CP: Carboplatin–Paclitaxel
CV: Cardiovascular
ER: Emergency room
HA: Headache
HR: Hazard ratio
HTN: Hypertension
NSCLC: Non–small cell lung cancer
OC: Ovarian cancer
OS: Overall survival
PFS: Progression-free survival
PO: Orally
QD: Daily
SBP: Systolic blood pressure
SOB: Shortness of breath
VDA: Vascular-disrupting agent
